# EHLS at School: school-age follow-up of the Early Home Learning Study cluster randomized controlled trial

**DOI:** 10.1186/s12887-018-1122-y

**Published:** 2018-05-02

**Authors:** Elizabeth M. Westrupp, Clair Bennett, Meabh Cullinane, Naomi J. Hackworth, Donna Berthelsen, Sheena Reilly, Fiona K. Mensah, Lisa Gold, Shannon K. Bennetts, Penny Levickis, Jan M. Nicholson

**Affiliations:** 1 Judith Lumley Centre, La Trobe University, Level 3, George Singer Building, Bundoora, Bundoora, VIC 3083 Australia; 2 Murdoch Children’s Research Institute, Melbourne, Australia; 3 Department of Paediatrics, The University of Melbourne, Melbourne, Australia; 4 School of Early Childhood and Inclusive Education, Queensland University of Technology, Brisbane, Australia; 5 Griffith University, Gold Coast, QLD Australia; 6 The Royal Children’s Hospital, Melbourne, Australia; 7 School of Health and Social Development, Deakin University, Geelong, Australia; 8 School of Education, Communication & Language Sciences, Newcastle University, Newcastle upon Tyne, UK

**Keywords:** School-age assessment, Long-term follow-up, Home learning environment, Cohort study, Education, Early childhood, Parenting

## Abstract

**Background:**

Targeted interventions during early childhood can assist families in providing strong foundations that promote children’s health and wellbeing across the life course. There is growing recognition that longer follow-up times are necessary to assess intervention outcomes, as effects may change as children develop. The Early Home Learning Study, or ‘EHLS’, comprised two cluster randomized controlled superiority trials of a brief parenting intervention, *smalltalk*, aimed at supporting parents to strengthen the early childhood home learning environment of infants (6–12 months) or toddlers (12–36 months). Results showed sustained improvements in parent-child interactions and the home environment at the 32 week follow-up for the toddler but not the infant trial. The current study will therefore follow up the EHLS toddler cohort to primary school age, with the aim of addressing a gap in literature concerning long-term effects of early childhood interventions focused on improving school readiness and later developmental outcomes.

**Methods:**

‘EHLS at School’ is a school-aged follow-up study of the toddler cluster randomized controlled trial (*n* = 1226). Data will be collected by parent-, child- and teacher-report questionnaires, recorded observations of parent-child interactions, and direct child assessment when children are aged 7.5 years old. Data linkage will provide additional data on child health and academic functioning at ages 5, 8 and 10 years. Child outcomes will be compared for families allocated to standard/usual care (control) versus those allocated to the *smalltalk* program (group program only or group program with additional home coaching).

**Discussion:**

Findings from The Early Home Learning Study provided evidence of the benefits of the *smalltalk* intervention delivered via facilitated playgroups for parents of toddlers. The EHLS at School Study aims to examine the long-term outcomes of this initiative to determine whether improvements in the quality of the parent-child relationship persist over time and translate into benefits for children’s social, academic and behavioral skills that last into the school years.

**Trial registration:**

8 September 2011; ACTRN12611000965909 (for the original EHLS)

## Background

In Australia, 23% of children entering primary school have failed to acquire the developmental skills essential for success in the school environment [1]. Children from socially and economically disadvantaged families are particularly vulnerable to poorer development of early socio-emotional, language and cognitive skills [2–8]. Early childhood is a key period in which targeted interventions can support families in providing their children with strong foundations for health and wellbeing across the life course [9, 10].

Achievement of good developmental outcomes by disadvantaged children is associated with a rich home learning environment in early childhood [1, 11]. These environments are characterized by frequent positive parent-child interactions, such as verbal exchanges, where parents respond to and build on the child’s interests, and use books, toys and other materials to extend the child’s developing skills [12]. The quality of the home environment exerts strong and lasting effects on children’s development, independently of other community or educational factors or interventions [13–17]. The United Kingdom Effective Pre-School, Primary and Secondary Education Project (1997–2013, N ≈ 3000) found that the quality of the home learning environment at age 3 predicted children’s reading and numeracy at ages 5 and 7 years (effect sizes from .50 to .73), and their literacy, English, mathematics and science attainment at age 10 (effect sizes from .29 to.49). The effects of the home learning environment were stronger than socio-economic status, parent education or income (at 3, 5 and 7), and the quality of pre-school and primary school experiences at age 10 [11].

Effective intervention to promote an enriched home learning environment for vulnerable children is hampered by a lack of evidence. Interventions to enhance the learning environment of childcare settings have shown benefits [18, 19]. Only a few studies internationally have sought to enhance the quality of the learning environment at home, and while promising, these are limited by small sample sizes and short-term follow-ups [20, 21]. In Australia, we are aware of two programs in use that seek to enhance the home learning environment [22, 23]. Neither has been the subject of a rigorous evaluation using a randomized controlled trial design and the long-term effects on the home learning environment and children’s outcomes are unknown.

The Early Home Learning Study (EHLS, 2009–2013) [24] was a community-based effectiveness trial of *smalltalk*, a new program designed to help parents in disadvantaged circumstances provide an enriched home environment to enhance the development of their young children (aged 6–36 months). The *smalltalk* program was developed in partnership with the State Government of Victoria, Australia, and was evaluated by the EHLS [25]. The EHLS comprised two cluster randomized controlled trials conducted in parallel, each delivered by a non-specialist workforce within one of two existing services: a 6-week maternal and child health parenting group program for parents of infants aged 6–12 months, and a 10-week facilitated playgroup program for parents of toddlers aged 12–36 months.

Twenty two local government authorities participated across the two trials, with up to six community locations (clusters) nominated for program delivery within each. Clusters were randomly allocated to one of three trial conditions: intervention (*smalltalk group-only*); enhanced intervention with home coaching (*smalltalk plus*); and ‘standard care’/usual practice (control). Participants received the intervention that was offered by the location servicing their geographic region. Between 2010 and 2012, programs were delivered to 2228 families attending 389 groups in 101 sites with 94 staff trained to provide either standard or *smalltalk* programs (not both). Reach, retention, satisfaction and program fidelity were good to excellent [26]. Eighty-four percent of referred families agreed to participate. Across the three conditions, participants attended an average of 59–64% of group sessions. Further details of the intervention format and delivery are listed elsewhere [24].

Parent-child interactions, the home environment and children’s communication and social skills were assessed at baseline (0 weeks), post-intervention (12 weeks) and follow-up (32 weeks) [25]. In the infant trial, there were no differences by trial arm on the primary outcomes at 32 weeks. In contrast, in the toddler trial, consistent benefits of the *smalltalk* program were evident at 32 weeks, including parent-reported improvements in the home learning environment (effect sizes 0.16 to 0.17), and most substantively, improvements in the directly observed parent-child interactions (effect sizes 0.46 to 0.55) [25]. Other findings included positive effects on child development for toddlers, with a 37% reduced odds of poor child social skills for *smalltalk* vs. control; and consistently greater gains for families receiving home coaching (i.e., ‘*smalltalk plus’*) compared to the *smalltalk group-only* program (unpublished findings, available on request) [26]. Given these results, the current study aims to follow participants from the toddler trial (and not the infant trial) to school age. In the absence of longer-term outcome data for the toddler cohort, it is unknown whether early gains will translate into improved developmental skills for children at school age, with ongoing health, social and economic benefits.

The skills that children bring with them as they enter primary school lay critical foundations for subsequent behavioral, social and academic skills [10]. The previous research findings from the EHLS indicated that families can be supported to enrich the home environment for toddlers from disadvantaged backgrounds with a fairly ‘light touch’, brief community-based intervention, yet we do not know if these effects are sustained sufficiently to improve school readiness and later developmental outcomes at school. Follow-up of the EHLS toddler cohort in early primary school will address a major gap in current evidence, and will provide foundations for better targeting of early interventions to support children at risk of poor developmental outcomes. There is growing recognition that long follow-up times are necessary as intervention effects may change as children develop [17], and the deleterious effects of social disadvantage tend to become stronger over time [18, 27]. Internationally, there is limited prospective evidence regarding the long-term effectiveness of early interventions, and particularly limited knowledge of the effects of intervention on vulnerable children under five [28]. Our proposed school-age assessment will address this gap and provide robust evidence of the extent to which an early childhood home learning intervention helps to reduce socio-economic inequalities in early child development.

The Differential Susceptibility Hypothesis [29–31] suggests that some children and parents are more sensitive to specific environmental experiences, and thus to intervention, than others. These effects are not identified using the traditional evaluation approach of averaging intervention effects, given that some parents and children may respond well or very well, while others are less affected. A school-age assessment of the large EHLS cohort also provides an opportunity to evaluate children’s sensitivity to changes in parenting and the home learning environment and to map the differential effects of the *smalltalk* intervention on child outcomes, in the context of (a) individual factors (e.g. child self-regulation), and (b) other parent and family contextual factors (e.g. parent mental health, adverse life events).

## Methods/design

### Aims

This follow-up study, EHLS at School, is focused on the toddler cohort who participated in the facilitated playgroup intervention arm. The decision to limit the school-age follow-up to the toddler cohort was required by resource availability that limited follow-up to one intervention arm of the EHLS. Findings from the EHLS indicated that substantive gains from the *smalltalk* intervention were observed in the toddler trial (parents of toddlers 12–36 months), compared to the infant cohort where gains were not maintained. The study aims are to:

1. investigate the effects of the toddler parenting program *smalltalk* on children’s school readiness, social and emotional development, and academic functioning;
2. investigate whether initial gains in parent-child interactions and the home environment are maintained to age 7.5;
3. identify the program attributes (program intensity, quality and timing) associated with gains in parent-child interactions, the home environment and child outcomes;
4. examine whether the benefits of *smalltalk* are affected by individual child and family factors such as self-regulation, and parent and family contextual factors; and
5. determine the financial, health and education costs and cost-effectiveness of *smalltalk*.

### Study design

The EHLS at School Study is a prospective cohort study involving the school-age follow-up of the EHLS toddler cohort (aged 12–36 months at baseline), with direct data collection from participants occurring when children are 7.5 years old. Additional outcome data will be available via data linkage when children are aged 5, 8 and 10 years. Data collection for the study commenced in early 2016 and is expected to continue until December 2018. Participants in the *smalltalk* and *smalltalk plus* groups will be analyzed together, and are subsequently referred to as simply ‘*smalltalk*’. Note that all research staff are blinded to participants’ original allocation to the *smalltalk* or standard group conditions for all aspects of data collection.

### Eligibility criteria

**Fig. 1:**
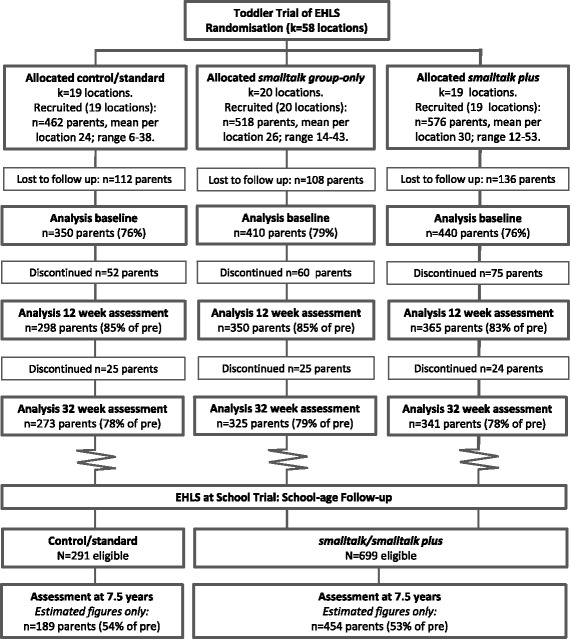
Participant flow in the Early Home Learning Study and estimated follow-up in the EHLS at School Study

For the current study, participants in the toddler trial (*n* = 1226) who completed an assessment prior to, and post intervention, or at the 32 week follow-up (the majority of participants completed both assessments; 93%) were re-contacted for participation. Participants who declined to be contacted for future research or those who actively withdrew from the EHLS were excluded. Participants were identified as eligible for the original EHLS study if the family had a child in the target age range and met at least one risk factor for socio-economic disadvantage (i.e., parent < 25 yrs., low parent education, single parent, low family income, Aboriginal or non-English speaking background). Families with insufficient oral English to complete assessments or families who would benefit from more intensive support services were excluded. Sample characteristics from the EHLS are documented elsewhere [25]. Participant flow in the EHLS and estimated follow-up participation in EHLS at School are shown in Fig. 1.

### Power calculation

Given the high rate of retention in the EHLS (78% to 32 week follow-up), we anticipate 65% participation in EHLS at School of eligible participants (i.e., *n* = 212 standard; *n* = 455 *smalltalk* participants). For the comparison of the *smalltalk* to standard programs, the study provides over 95% power to detect a minimum effect size of 0.3, or 88% power to detect an effect size of 0.25, at *p* < 0.05.

### Recruitment strategy

In EHLS at School, children are eligible to be assessed when they reach 7.5 years of age. Given that toddlers in the original EHLS were aged 12–36 months at baseline assessment over 2010–2013, children are eligible for the EHLS at School Study over a three-year period between 2016 and 2018. Recruitment is currently underway. At study commencement in 2016, all eligible participants (*n* = 990) were sent an information pack describing the study. The pack contained an information letter, contact details form, brochure describing the EHLS at School Study, EHLS at School fridge magnet, flyer describing the findings from the original EHLS Study, and reply paid envelope. Participants were invited to update their contact details via post, email, or phone. Following the mail out, the study team attempted to contact all participants via phone. If participants had already updated their contact details, the purpose of the phone call was to select an age-appropriate children’s book to be posted to the study child. Otherwise, the phone call was used to introduce the study and update participant details (and subsequently arrange for the book to be posted).

Participants are currently being re-contacted prior to the age of eligibility (7.5 years) to arrange and conduct a home visit assessment. Participants who have moved interstate, overseas or who declined a home visit were offered an online or hardcopy option for completing the questionnaire aspects of the assessment. All participants received a $50 gift voucher on completion of the home assessment, or alternatively, a $30 voucher on completion of the parent questionnaire only. To maintain engagement, study children who have not yet been assessed are sent a birthday card each year. Following the home visit or online assessment, and with written parental consent, school teachers are contacted via email and/or phone and invited to complete an online questionnaire.

### Outcome measures

To address Aims 1 and 3–5, assessed child outcomes include school readiness, child socio-emotional development, language, mental health, self-regulation and physical health, and academic functioning. To address Aim 2, the home environment and parent-child interactions are assessed. Table 1 provides a summary of domains assessed across the EHLS and EHLS at School studies, while Table 2 provides a detailed summary of key measures included in EHLS at School.

**Table 1:** Measurement domains assessed in the EHLS and the EHLS at School studies

Domain	Outcome	Data collection method	EHLS			EHLS at School		
			Pre	Po	FU	5y	7.5y	8-10y
*Child and Parent Outcomes*								
Child language and academic	Language, communication	PQ DA DL	✓	✓	✓	✓	✓	✓
	School readiness, English skills	DL				✓		
	Academic skills	TQ DL					✓	✓
Child socio-emotional and general development	Fine motor skills	PQ	✓	✓	✓			
	Personal-social skills	PQ	✓	✓	✓			
	Developmental status	DL				✓		
	Mental health problems, quality of life	PQ CQ TQ DL				✓	✓	
	Social skills	PQ					✓	
	Self-regulation	PQ					✓	
Parenting and home environment	Parent-child interaction	DA	✓	✓	✓		✓	
	Parenting irritability, warmth, consistency, self-efficacy, verbal responsivity	PQ	✓	✓	✓		✓	
	Home learning activities; home literacy environment	PQ	✓	✓	✓		✓	
*Intervention Experiences*								
*smalltalk* exposure	Number of sessions attended, parent engagement, content exposure	Adm		✓				
Other services	Number and type of sessions attended	PQ					✓	
*Covariates*								
Child	Health and disability, temperament	PQ TQ	✓	✓	✓		✓	
Parent	Mental health, quality of life	PQ	✓	✓	✓		✓	
Family	Demographics, family structure, stressful events, social support	PQ	✓	✓	✓		✓	
Services	Use of educational services and child care	PQ	✓	✓	✓		✓	

**TABLE KEY:** *Pre* Pre assessment, *Po* Post assessment, *FU* Follow-up assessment, *PQ* parent-report questionnaire, *CQ* child-report questionnaire, *DA* direct (child) assessment, *DL* data linkage, *TQ* teacher questionnaire, *Adm* administrative data from group facilitators/home coaches

**Table 2:** Summary of key study measures used in the EHLS at School study

Construct	Measurement Details	Data Collection Method and Age
*Child Development*		
School readiness	English Online Interview [44] is administered by teachers at the beginning of the Preparatory/Foundational Year, taking 20–40 min to complete and assessing children’s reading, writing and speaking, and listening skills.	Data linkage to direct teacher assessment of child (5 years)
	School Entrant Health Questionnaire [41] collects data on parents’ concerns and observations about their child’s language, health and wellbeing.	Data linkage to parent-report questionnaire (5 years)
Language	National Institutes of Health Toolbox Picture Vocabulary Test [33] assessing receptive vocabulary.	Direct child assessment (7.5 years)
	Recalling Sentences subtest of the Clinical Evaluation of Language Fundamentals - Fourth Edition [34]; the child is asked to imitate sentences spoken aloud by the researcher.	Direct child assessment (7.5 years)
	Castles and Coltheart Test of Reading Version 2 [35] measuring children’s sounding out ability and whole word recognition.	Direct child assessment (7.5 years)
Academic skills	National Assessment Program – Literacy and Numeracy [45] assesses children’s reading, numeracy, spelling, punctuation and grammar.	Data linkage to national assessment data for Year 3 (age 8) and 5 (age 10)
	Academic Rating Scale [46] assesses children’s academic achievement in terms of language and literacy (10 items) and mathematical ability (8 items).	Teacher questionnaire (7.5 years)
Mental health	Strengths and Difficulties Questionnaire [42] assesses emotional and behavioral problems across 5 subscales (25 items) including hyperactivity/inattention; conduct problems; emotional symptoms, peer problems; and prosocial skills.	Data linkage to parent-report questionnaire (5 years); Parent questionnaire (7.5 years); Teacher questionnaire (7.5 years)
Self-regulation	Approaches to Learning Scale [47] (6 items) assesses children’s eagerness to learn, interest in a variety of things, creativity, persistence, concentration and responsibility, and is rated on a 4 point scale from ‘Never’ to ‘Very often’ or rated as ‘No opportunity to observe this behavior.’	Parent questionnaire (7.5 years); Teacher questionnaire (7.5 years)
	Soothability and Inhibitory Control subscales from the Temperament in Middle Childhood Questionnaire [48] (16 items) assesses children’s rate of recovery from peak distress, excitement, or general arousal, and their capacity to plan and to suppress inappropriate approach responses under instructions or in novel or uncertain situations.	Parent questionnaire (7.5 years)
Quality of life	The Child Health Utility 9D [36] assesses children’s health-related quality of life using 9 questions rated on a 5-point scale. This measure was developed for the use in cost utility analysis (i.e., economic evaluation).	Child assessment (7.5 years)
Physical health	Child global health [49] (1 item) is rated on a 5-point scale from 1 = ‘Excellent’ to 5 = ‘Poor’.	Parent questionnaire (7.5 years)
	Child Health History (3 items) asks parents about hospital stays over past 12 months; current presence of 10 health problems, e.g., ‘wheezing or asthma’; ‘snoring or difficulty sleeping’; and whether ever diagnosed or treated for psychological disorder.	Parent questionnaire (7.5 years)
	Use of health services (1 item) asks parents whether they have used specific health services for their child (e.g., family doctor; Pediatrician; psychological services; dentist), and the cost to the family per visit.	Parent questionnaire (7.5 years)
*Parent-Child Relationship*		
Parent-child interactions	The Sensitive Responding and Mutuality subscales of the Coding of Attachment-Related Parenting scheme [40] assesses attachment-related parenting behaviors rated on a 7-point Likert scale 1 = ‘No evidence’; 7 = ‘Pervasive/extreme evidence’.	Direct child assessment (7.5 years)
Parenting	Parenting warmth [49] (6 items) is rated on a 5-point scale, e.g., “Thinking about the last 6 months, how often do you… hug or hold your child?”	Parent questionnaire (7.5 years)
	Parenting irritability [49] (5 items) is rated on a 5-point scale, e.g., “Thinking about the last 4 weeks, how often have you… lost your temper with your child?”	Parent questionnaire (7.5 years)
	Parenting consistency [49] (5 items) is rated on a 5-point scale, e.g., “When you discipline this child, how often does he/she ignore the punishment?”	Parent questionnaire (7.5 years)
	Parent self-efficacy [49] (4 items) is rated on a 5-point scale, e.g., “How often do you think that this child’s behavior is more than you can handle”.	Parent questionnaire (7.5 years)
	Global parenting self-efficacy [49] (1 item) is rated on a 5-point scale from 1 = “Not very good at being a parent” to 5 = “A very good parent”.	Parent questionnaire (7.5 years)
*Home Environment*		
Home learning activities	Home activities with child (5 items) [47] is rated on a 4-point scale assessing parental engagement of child in home activities that stimulate development, e.g., “Read books to your child”.	Parent questionnaire (7.5 years)
	Home Literacy Environment Scale (6 items) [50] is rated on various scales, e.g., “How many books does your child own?”	Parent questionnaire (7.5 years)
Disorganization	Confusion, Hubbub and Order Scale (6 items) [51] is rated on a yes/no scale, e.g., “The atmosphere in our home is calm”.	Parent questionnaire (7.5 years)
*Parent-Focused Outcomes*		
Psychological distress	Kessler-6 (6 items) [52] rated on a 5-point scale, assesses emotional distress over the past four weeks, e.g., “About how often did you feel nervous?”	Parent questionnaire (7.5 years)
Positive affect	Positive and Negative Affect Schedule, Short Form (5 items) [53].	Parent questionnaire (7.5 years)
Quality of life	The adult version of the Child Health Utility 9D [54].	Parent questionnaire (7.5 years)
*Covariates*		
Demographics	Parent and child age, ethnicity, language spoken, education, income, employment status, family structure and size.	Parent questionnaire (7.5 years)
	Characteristics of the child’s teacher, classroom and school, school culture, physical education, family engagement in school activities; child’s specialized services/needs, absenteeism.	Teacher questionnaire (7.5 years)
Contextual factors	Stressful life events (9 items) [55] over the past 12 months, e.g., “You became pregnant or had a baby; You moved house”.	
	Social support (1 item) is rated on a 4-point scale; 1 = ‘I get enough help’ to 3 = ‘I don’t get any help at all’, and − 1 = ‘I don’t need any help’.	
Student Teacher Relationship	The Pianta Student Teacher Relationship Scale [54] assesses teacher’s perception of teacher-child interactions, including warmth, irritability, compliance, and communication (15 items).	Teacher questionnaire (7.5 years)

Multi-method data collection for the EHLS at School study includes a home visit assessment and an online teacher questionnaire at 7.5 years. The home visit involves completion of a parent questionnaire, parent-child observation, and direct child assessment. Also at the 7.5 year home visit, parent permission is obtained for data linkage to retrospective state educational records of school readiness and academic skills at age 5 years, and prospective national academic test results for assessments conducted when children are in Year 3 (age 8) and Year 5 (age 10) of school. Where possible, measures are consistent across the different methods of assessment. For example, the Strengths and Difficulties Questionnaire, a measure of child socio-emotional development, will be accessed via data linkage to provide parent-report data on child outcomes at age 5, and is also collected at age 7.5 years using parent- and teacher-report questionnaires. Study data are collected and managed using REDCap electronic data capture tools hosted at La Trobe University and the Murdoch Childrens Research Institute (Melbourne, Australia). REDCap (Research Electronic Data Capture) is a secure, web-based application designed to support data capture for research studies [32].

### Direct child assessment measures (home assessment)

The following measures form the direct child assessment and are administered by a researcher during the home visit using an iPad to display or code the child responses.

#### Child language short form

This measure was developed as an iPad application as part of the Centre for Research Excellence in Child Language based at the Murdoch Childrens Research Institute in Melbourne, Australia, to enable population studies to cheaply, reliably and rapidly obtain a measure of language ability. The iPad application has two components: (1) the standardized ‘National Institutes of Health (NIH) Toolbox Picture Vocabulary Test’ [33], which was modified with permission from the United States NIH in order to be delivered on an iPad in an Australian accent, and (2) the standardized Recalling Sentences subtest of the Clinical Evaluation of Language Fundamentals - Fourth Edition [34] (NCS Pearson, Inc. - Reproduced by Permission).

#### Castles and Coltheart test of reading version 2

This is an Australian standardized measure assessing children’s sounding-out ability and whole word recognition in single word reading of regular words (40 items); irregular words (40 items); and non-words (40 items) [35].

#### Child health utility 9D

This 9-item child self-report questionnaire was developed to assess children’s health related quality of life for use in economic evaluation [36].

#### Temperament in middle childhood - self-report questionnaire

The Soothability and Inhibitory Control subscales (16 items) will be used to measure child self-regulation [37].

### Recorded parent-child observation (home assessment)

Direct observation is the gold standard for assessing the nature of parent-child interactions [38, 39]. At the home visit, the participating parent and study child are invited to take part in a free-play activity (5–10 min) and tidy-up task (5 min) using a set of age-appropriate toys and games provided (i.e., LEGO Classic Creative Bricks, Jenga, and animal snap cards). Both tasks are recorded by a researcher using an iPad and will subsequently be coded using the Coding of Attachment-Related Parenting (CARP) scheme [40]. The CARP is a behavioral observation system used to assess two dimensions of attachment-related parenting behaviors (‘Sensitive Responding’ and ‘Mutuality’) rated on a 7-point Likert scale (1 = No evidence; 7 = Pervasive/extreme evidence). *Sensitive Responding* assesses the degree to which the parent promotes their child’s autonomy, shows awareness and sensitivity to the child’s needs and signals, adopts the child’s perspective, and expresses verbal and physical warmth. *Mutuality* reflects the degree to which the parent-child dyad reciprocates positive affectionate behaviors, seeks joint engagement in an activity, maintains shared attention and affect sharing, and maintains physical proximity in interactions. Interrater reliability will be assessed for 10–20% of the participants.

### Parent-report questionnaire (home assessment or online)

Parents are invited to complete a questionnaire on an iPad using the REDCap application during the time that the researcher is conducting the direct child assessment in the home visit. The questionnaire takes parents about 30 min to complete, and consists of 9 sections, asking about parent characteristics (5 items), the study child’s early education experiences (4 items) and formal schooling (9 items), child behavior and development (47 items), child health and wellbeing (32 items), parenting and parent-child interactions (27 items), parent health (20 items), the home environment (11 items), and parent and family demographic information (15 items). Refer to Table 2 for more information regarding these measures.

### Data linkage

Data linkage to state and federal government datasets will be undertaken. Separate consent is requested from parents for each aspect of data linkage.

#### School entrant health questionnaire

This measure is completed by parents of children in government schools in the State of Victoria in Australia at school entry when children are approximately 5 years old [41]. The questionnaire records parents’ concerns and observations about their child’s health and wellbeing, and comprises a number of validated measures, including the Strengths and Difficulties Questionnaire [42] and the Parental Evaluation of Developmental Status [43]. The measure also has questions on speech and language, service use, and family issues and stressors.

#### English online interview

Teachers in Victorian government schools are mandated to assess all students at the beginning of their ‘Preparatory’ or ‘Foundation’ year (age 5) using the English Online Interview, to assess students’ reading, writing and speaking, and listening skills. The assessment consists of a one-to-one interview between a teacher and a student using purpose-developed texts and resources [44]. During the interview with the child, teachers enter scores directly into the online system, where the scores are automatically aggregated and converted to a scale score based on the Victorian Curriculum.

#### National Assessment Program – Literacy and numeracy (NAPLAN)

NAPLAN is an annual national assessment conducted for all Australian children in Years 3, 5, 7 and 9 of school. It is designed to assess children’s skills in a variety of domains including reading, numeracy, spelling, punctuation and grammar [45]. In the current study, we have approval from the Victorian Curriculum and Assessment Authority to link to NAPLAN data collected in Years 3 (age 8) and 5 (age 10).

### Teacher-report questionnaire (online)

Separate consent is requested from parents to allow research staff to contact the child’s current school teacher. The 15-min teacher questionnaire is administered online using the REDCap web application. The questionnaire consists of six sections asking teachers about the school and the child’s year level and classroom (13 items), the child’s and their family’s engagement with the school (10 items), the child’s academic performance (18 items), and social and behavioral development (31 items), the teacher-child relationship (15 items), and the teachers’ level of experience and the school environment (12 items). Items from the questionnaire have been modified from the teacher questionnaire used in the Longitudinal Study of Australian Children when children were 6–7 years of age.

### Statistical analyses

The planned analytic approach is ‘as-treated’ (rather than ‘intention to treat’) because families from control and *smalltalk* groups may have participated in *smalltalk* after completion of the EHLS, and a small number (*n* = 38) who were allocated to *smalltalk* completed assessments but did not attend any sessions. Therefore, the primary comparisons will be participants receiving any exposure to *smalltalk* compared to those participants receiving no exposure to *smalltalk*. Unadjusted and adjusted analyses will be conducted, the latter to account for differential characteristics of participants exposed and not exposed to *smalltalk* (e.g. child age, parent age and mental health, family demographic factors).

Data will be analyzed using Stata software. To address Aim 1, linear and logistic regression analyses will test the effect of exposure to *smalltalk* versus no exposure in determining child outcomes via unadjusted and adjusted analyses. To address Aim 2, linear regression analyses will investigate whether early improvements in parent-child interactions associated with *smalltalk* were sustained to 7.5 years. Sub-group analyses will examine whether child and family characteristics predict sustained improvements, i.e., for families experiencing multiple social and economic risks. We will also test whether sustained improvements in parent-child interactions mediate the relationship between *smalltalk* exposure and child outcomes at ages 5, 7.5, 8, and 10 years.

To address Aim 3, we will use the rich process data collected during the original EHLS. We will test whether condition intensity (with vs. without home coaching), dosage (number of sessions attended), and parent engagement in the program sessions influence child outcomes. Regression analyses will be used to test for potential moderating effects of program-specific factors on the relationship between *smalltalk* exposure and parent and child outcomes. To address Aim 4, we will examine child, parent and environmental factors to determine which children are more or less likely to benefit from *smalltalk.* For example, we will examine the extent to which children with poor self-regulation skills, or from families facing multiple socio-economic disadvantages, show differential benefits from their parents’ participation in *smalltalk*. Regression analyses including interaction terms between intervention and child/family factors will be used to test whether child/family factors moderate intervention effects.

To address Aim 5, we will conduct an economic evaluation of the *smalltalk* program. Costs of *smalltalk* incurred by service providers and families from intervention to 7.5 years of age will be estimated from prospectively recorded data on resources used in the *smalltalk* program and parent-report of children’s use of health, social and educational services up to age 7.5 years. These costs will then be compared to the full range of outcomes detailed above using a cost-consequences analysis, which compares any additional costs of *smalltalk* (compared to controls) to the full range of change in outcomes achieved. Analyses will include extensive sensitivity testing to assess the impact of uncertainty in data and modelling assumptions on results.

### Progress

As of May 2017, the research team has attempted contact with all eligible participants (*n* = 990), of which 637 (64%) have agreed to further contact when their child reaches the eligible age, and 128 (13%) have declined or ‘passively refused’ (i.e., maximum attempts to contact reached with no participation). Further attempts to make contact will continue for the remaining 225 (23%) participants. Assessments have been completed for 373 participants. Of these, 16 participants did not complete the home assessment but instead provided data in the form of the online parent and teacher questionnaires, and consent for data linkage. So far, more than 90% of parents have consented to completion of the online teacher questionnaires, and to data linkage. Based on completed fieldwork, we anticipate a final sample with complete child and parent data of between 60% and 65% of the initial sample.

## Discussion

The original EHLS was ground breaking in a number of ways. It was the first Australian study and one of only a few internationally to employ rigorous program development and research methods to examine the effectiveness of a parent-focused early home learning intervention. Key strengths included: (1) a 9-month development process to ensure the evidence-informed intervention strategies were appropriate to target parents and could be delivered reliably within the existing service system; (2) application of a cluster-randomized controlled trial design, with strategies to minimize cross-condition contamination (site-based allocation to condition; staff trained in one condition only) and researcher bias (blinding of field/coding staff); and (3) the collection of data from a large sample, with excellent participation rates and retention over time, enhancing confidence in the generalizability of study findings [25].

The EHLS has already had a significant influence on early childhood policy in Victoria, Australia. On the basis of the evidence of *smalltalk*’s short-term benefits to socially and economically disadvantaged families, the State Government has invested in its state-wide roll-out with potential for widespread benefits to vulnerable children. While the EHLS findings are highly promising, they leave unanswered the question of whether *smalltalk* results in sustained changes in the home environment and enhancement of children’s capabilities by the time they enter school.

Internationally, economic evaluations of early life interventions are scarce, particularly those based on long-term follow-ups of rigorously implemented randomized controlled trials [28]. Despite this, there is evidence that early interventions which result in long-term gains for children have substantial economic benefits at a societal level [28]. We have good reason to expect that the modest short-term gains seen in the EHLS could translate into sizeable economic benefits if sustained over time. Our combined outcomes data and economic evaluation will provide important new knowledge to underpin the development of service systems effectively targeted to the needs of disadvantaged families.

### Study status

Ongoing.

## Abbreviations

Adm: Administrative data from group facilitators/home coaches
CARP: Coding of Attachment-Related Parenting
CQ: Child-report questionnaire
DA: direct (child) assessment
DET: Department of Education and Training
DL: Data linkage
EHLS: Early Home Learning Study
FU: Follow-up assessment
NAPLAN: National Assessment Program – Literacy and Numeracy
NIH: National Institutes of Health
Po: Post assessment
Pre: Pre assessment
PQ: Parent-report questionnaire
REDCap: Research Electronic Data Capture
TQ: Teacher questionnaire
